# Supplementary material for: Improved estimates for extinction probabilities and times to extinction for populations of tsetse (Glossina spp)
Source: PLoS Negl Trop Dis. 2019 Apr 9;13(4):e0006973. doi: 10.1371/journal.pntd.0006973 (PMC6474634; doi:10.1371/journal.pntd.0006973)
Supplement: S1 Text — (PDF) [file pntd.0006973.s001.pdf]

Improved estimates for extinction probabilities and times to extinction for populations of tsetse (*Glossina* spp)

Damian Kajunguri[1], Elisha B. Are[2], John W. Hargrove[2].

1 Department of Mathematics, Kabale University, Kabale, Uganda.
2 Centre of Excellence in Epidemiological Modelling and Analysis (SACEMA), University of Stellenbosch, Stellenbosch, South Africa.

P. O. Box 317, Kabale, Uganda.
dkajunguri@kab.ac.ug

S1 Text

Proof of equation (7)

When $k = 0$, we obtain

$$\begin{aligned} p_0 &= \epsilon\lambda^{\nu}(1-\lambda^{\tau})\sum_{n=0}^{\infty}\binom{n}{0}(\lambda^{\tau}\beta)^n(\frac{1}{\beta}-\varphi^P))^n \\ &= \epsilon\lambda^{\nu}(1-\lambda^{\tau})\sum_{n=0}^{\infty}[\lambda^{\tau}(1-\beta\varphi^P))]^n \\ &= \epsilon\lambda^{\nu}(1-\lambda^{\tau})\left[\frac{1}{(1-\lambda^{\tau}(1-\beta\varphi^P)))}\right] \\ &= \frac{\epsilon\lambda^{\nu}(1-\lambda^{\tau})}{(1-\lambda^{\tau}(1-\beta\varphi^P)))}. \end{aligned}$$

When $k = 1$, we obtain

$$\begin{aligned} p_1 &= \epsilon\lambda^{\nu}(1-\lambda^{\tau})\varphi^P\sum_{n=1}^{\infty}\binom{n}{1}(\lambda^{\tau}\beta)^n(\frac{1}{\beta}-\varphi^P)^{n-1} \\ &= \epsilon\lambda^{\nu}(1-\lambda^{\tau})\varphi^P\sum_{n=1}^{\infty}n(\lambda^{\tau}\beta)^n(\frac{1}{\beta}-\varphi^P))^{n-1}. \end{aligned}$$

If we let $a = \lambda^{\tau}\beta, b = (\frac{1}{\beta}-\varphi^P))$ and $F = \sum_{n=1}^{\infty} na^nb^{n-1}$, this implies that

$$\begin{aligned} F &= ab^0 + 2a^2b + 3a^3b^2 + \dots \\ abF &= a^2b + 2a^3b^2 + \dots \\ (1-ab)F &= ab^0 + a^2b + a^3b^2 + \dots \\ ab(1-ab)F &= a^2b + a^3b^2 + a^4b^3 + \dots = \frac{a^2b}{(1-ab)} \\ F &= \frac{a}{(1-ab)^2} = \frac{\lambda^{\tau}\beta}{(1-\lambda^{\tau}(1-\beta\varphi^P)))^2}. \end{aligned}$$

Thus, the final solution for p_1 becomes

$$\begin{aligned} p_1 &= \epsilon\lambda^{\nu}(1-\lambda^{\tau})\varphi^{P}F \\ &= \epsilon\lambda^{\nu}(1-\lambda^{\tau})\varphi^{P}\left[\frac{\lambda^{\tau}\beta}{(1-\lambda^{\tau}(1-\beta\varphi^{P}))^2}\right] \\ &= \frac{\epsilon\lambda^{\nu+\tau}(1-\lambda^{\tau})\beta\varphi^{P}}{(1-\lambda^{\tau}(1-\beta\varphi^{P})))^2}. \end{aligned}$$

When $k=2$, we obtain

$$\begin{aligned} p_2 &= \epsilon\lambda^{\nu}(1-\lambda^{\tau})\varphi^{2P}\sum_{n=2}^{\infty}\binom{n}{2}(\lambda^{\tau}\beta)^n(\frac{1}{\beta}-\varphi^{P}))^{n-2} \\ &= \epsilon\lambda^{\nu}(1-\lambda^{\tau})\varphi^{2P}\sum_{n=2}^{\infty}\left[\frac{n(n-1)}{2}(\lambda^{\tau}\beta)^n(\frac{1}{\beta}-\varphi^{P}))^{n-2}\right]. \end{aligned}$$

Also letting $a=\lambda^{\tau}\beta$, $b=(\frac{1}{\beta}-\varphi^{P})$ and $G=\sum_{n=2}^{\infty}[\frac{n(n-1)}{2}a^nb^{n-2}]$, we have

$$\begin{aligned} G &= a^2b^0+3a^3b+6a^4b^2+10a^5b^3+\ldots \\ abG &= a^3b+3a^4b^2+6a^5b^3+\ldots \\ (1-ab)G &= a^2b^0+2a^3b+3a^4b^2+4a^5b^3+\ldots \\ ab(1-ab)G &= a^3b+2a^4b^2+3a^5b^3+4a^6b^4+\ldots \\ ((1-ab)-ab(1-ab))G=(1-ab)^2G &= a^2b^0+a^3b+a^4b^2+a^5b^3+\ldots \\ ab(1-ab)^2G &= a^3b+a^4b^2+a^5b^3+a^6b^4+\ldots=\frac{a^3b}{(1-ab)}, \\ G &= \frac{a^2}{(1-ab)^3}=\frac{(\lambda^{\tau}\beta)^2}{(1-\lambda^{\tau}(1-\beta\varphi^{P}))^3}. \end{aligned}$$

Thus, the final solution for p_2 becomes

$$\begin{aligned} p_2 &= \epsilon\lambda^{\nu}(1-\lambda^{\tau})\varphi^{2P}G \\ &= \epsilon\lambda^{\nu}(1-\lambda^{\tau})\varphi^{2P}\left[\frac{(\lambda^{\tau}\beta)^2}{(1-\lambda^{\tau}(1-\beta\varphi^{P}))^3}\right] \\ &= \frac{\epsilon\lambda^{\nu+2\tau}(1-\lambda^{\tau})\beta^2\varphi^{2P}}{(1-\lambda^{\tau}(1-\beta\varphi^{P})))^3}. \end{aligned}$$

Thus, in general

$$p_k=\frac{\epsilon\lambda^{\nu+k\tau}(1-\lambda^{\tau})\beta^k\varphi^{kP}}{(1-\beta\lambda^{\tau}(\frac{1}{\beta}-\varphi^{P}))^{k+1}},\quad for\quad k>0.$$

Proof of equations (8) and (10)

$$\begin{aligned}
p_{(k>0)} &= \sum_{k=1}^{\infty} \frac{\epsilon\lambda^{\nu+k\tau}(1-\lambda^{\tau})\beta^{k}\varphi^{kP}}{(1-\beta\lambda^{\tau}(\frac{1}{\beta}-\varphi^{P}))^{k+1}} \\
&= \frac{\epsilon\lambda^{\nu}(1-\lambda^{\tau})}{1-\lambda^{\tau}(1-\beta\varphi^{P})} \sum_{k=1}^{\infty} \left[\frac{\lambda^{\tau}\beta\varphi^{P}}{1-\lambda^{\tau}(1-\beta\varphi^{P})}\right]^{k} \\
&= \frac{\epsilon\lambda^{\nu}(1-\lambda^{\tau})}{1-\lambda^{\tau}(1-\beta\varphi^{P}))} \left[\frac{\frac{\lambda^{\tau}\beta\varphi^{P}}{1-\lambda^{\tau}(1-\beta\varphi^{P})}}{1-\frac{\lambda^{\tau}\beta\varphi^{P}}{1-\lambda^{\tau}(1-\beta\varphi^{P})}}\right] \\
&= \frac{\epsilon\lambda^{\nu}(1-\lambda^{\tau})}{1-\lambda^{\tau}(1-\beta\varphi^{P})} \left[\frac{\lambda^{\tau}\beta\varphi^{P}}{1-\lambda^{\tau}(1-\beta\varphi^{P}))-\lambda^{\tau}\beta\varphi^{P}}\right] \\
&= \frac{\epsilon\lambda^{\nu}(1-\lambda^{\tau})}{1-\lambda^{\tau}(1-\beta\varphi^{P})} \left[\frac{\lambda^{\tau}\beta\varphi^{P}}{(1-\lambda^{\tau})}\right] \\
&= \frac{\epsilon\lambda^{\nu+\tau}\beta\varphi^{P}}{1-\lambda^{\tau}(1-\beta\varphi^{P}))}.
\end{aligned}$$

Thus, the probability that a female tsetse fly does not produce any surviving female offspring before she dies is given by:

$$p_0 = 1 - p_{(k>0)} = 1 - \frac{\epsilon\lambda^{\nu+\tau}\beta\varphi^{P}}{1-\lambda^{\tau}(1-\beta\varphi^{P}))}.$$

Extinction probability, $\phi(\theta)$ is:

$$\phi(\theta) = \sum_{k=0}^{\infty} p_k\theta^k = p_0 + \sum_{k=1}^{\infty} p_k\theta^k.$$

$$\begin{aligned}
\phi(\theta) &= 1 - \frac{\epsilon\lambda^{\nu+\tau}\beta\varphi^{P}}{1-\beta\lambda^{\tau}(1+(1-\varphi^{P}))} + \frac{\epsilon\lambda^{\nu}(1-\lambda^{\tau})}{1-\lambda^{\tau}(\frac{1}{\beta}-\varphi^{P})} \sum_{k=1}^{\infty} \left[\frac{\lambda^{\tau}\beta\varphi^{P}\theta}{1-\lambda^{\tau}(\frac{1}{\beta}-\varphi^{P}))}\right]^{k} \\
&= 1 - \frac{\epsilon\lambda^{\nu+\tau}\beta\varphi^{P}}{1-\lambda^{\tau}(1-\beta\varphi^{P})} + \frac{\epsilon\lambda^{\nu}(1-\lambda^{\tau})}{1-\lambda^{\tau}(1-\beta\varphi^{P})} \left[\frac{\frac{\lambda^{\tau}\beta\varphi^{P}\theta}{1-\lambda^{\tau}(1-\beta\varphi^{P})}}{1-\frac{\lambda^{\tau}\beta\varphi^{P}\theta}{1-\lambda^{\tau}(1-\beta\varphi^{P})}}\right] \\
&= 1 - \frac{\epsilon\lambda^{\nu+\tau}\beta\varphi^{P}}{1-\lambda^{\tau}+\beta\lambda^{\tau}\varphi^{P}} + \frac{\epsilon\lambda^{\nu}(1-\lambda^{\tau})}{1-\lambda^{\tau}+\beta\lambda^{\tau}\varphi^{P}} \left[\frac{\lambda^{\tau}\beta\varphi^{P}\theta}{1-\lambda^{\tau}+\beta\lambda^{\tau}\varphi^{P}-\beta\lambda^{\tau}\varphi^{P}\theta}\right].
\end{aligned}$$

Setting $A = 1 - \lambda^\tau$, $B = \beta\lambda^\tau\varphi^P$ and $C = 1 - \epsilon\lambda^\nu$, we obtain

$$\begin{aligned}
\phi(\theta) &= 1 - \frac{\epsilon\lambda^\nu B}{A+B} + \frac{\epsilon\lambda^\nu A}{A+B}\left[\frac{B\theta}{A+B-B\theta}\right] \\
&= \frac{(A+B)(A+B-B\theta) - \epsilon\lambda^\nu B(A+B-B\theta) + \epsilon AB\lambda^\nu\theta}{(A+B)(A+B-B\theta)} \\
&= \frac{(A+B)(A+B-B\theta) - \epsilon\lambda^\nu B(A+B) + \epsilon\lambda^\nu B^2\theta + \epsilon\lambda^\nu AB\theta}{(A+B)(A+B-B\theta)} \\
&= \frac{(A+B)(A+B-B\theta) - (A+B)(\epsilon\lambda^\nu B) + (A+B)\epsilon\lambda^\nu B\theta}{(A+B)(A+B-B\theta)} \\
&= \frac{(A+B)(A+B-B\theta - \epsilon\lambda^\nu B + \epsilon\lambda^\nu B\theta)}{(A+B)(A+B-B\theta)} \\
&= \frac{A+B(1-\theta-\epsilon\lambda^\nu+\epsilon\lambda^\nu\theta)}{A+B(1-\theta)} \\
&= \frac{A+B(1-\theta)(1-\epsilon\lambda^\nu)}{A+B(1-\theta)} \\
&= \frac{A+BC(1-\theta)}{A+B(1-\theta)}.
\end{aligned}$$

Proof of equations (12) and (13)

$$\begin{aligned}
M_1 &= \sum_{k=0}^{\infty} kp_k \\
&= \sum_{k=0}^{\infty} \frac{k\epsilon\lambda^{\nu+k\tau}(1-\lambda^\tau)\beta^k\varphi^{kP}}{(1-\beta\lambda^\tau(\frac{1}{\beta}-\varphi^P))^{k+1}} \\
&= \frac{\epsilon\lambda^\nu(1-\lambda^\tau)}{1-\beta\lambda^\tau(\frac{1}{\beta}-\varphi^P)} \sum_{k=0}^{\infty} k\left[\frac{\lambda^\tau\beta\varphi^P}{1-\beta\lambda^\tau(\frac{1}{\beta}-\varphi^P)}\right]^k.
\end{aligned}$$

Using the sum of power series, that is $\sum_{n=0}^{\infty} nx^n = \frac{x}{(1-x)^2}$ to simplify the terms not involing the summation sign, we obtain

$$\begin{aligned}
M_1 &= \frac{\epsilon\lambda^\nu(1-\lambda^\tau)}{1-\lambda^\tau(1-\beta\varphi^P)}\left[\frac{\frac{\lambda^\tau\beta\varphi^P}{1-\lambda^\tau(1-\beta\varphi^P)}}{(1-\frac{\lambda^\tau\beta\varphi^P}{1-\lambda^\tau(1-\beta\varphi^P)})^2}\right] \\
&= \frac{\epsilon\lambda^\nu(1-\lambda^\tau)}{1-\lambda^\tau(1-\beta\varphi^P)}\left[\frac{\frac{\lambda^\tau\beta\varphi^P}{1-\lambda^\tau(1-\beta\varphi^P)}}{\frac{(1-\beta\lambda^\tau(1+(1-\varphi^P))+\lambda^\tau\beta\varphi^P)^2}{(1-\lambda^\tau(1-\beta\varphi^P))^2}}\right] \\
&= \frac{\epsilon\lambda^\nu(1-\lambda^\tau)}{1-\lambda^\tau(1-\beta\varphi^P)}\left[\frac{\lambda^\tau\beta\varphi^P(1-\lambda^\tau(1-\beta\varphi^P))}{(1-\lambda^\tau(1-\beta\varphi^P)-\lambda^\tau\beta\varphi^P)^2}\right] \\
&= \frac{\epsilon\lambda^{\nu+\tau}(1-\lambda^\tau)\beta\varphi^P}{(1-\lambda^\tau)^2} \\
&= \frac{\epsilon\lambda^{\nu+\tau}\beta\varphi^P}{(1-\lambda^\tau)}.
\end{aligned}$$

$$\begin{aligned} M_2 &= \sum_{k=0}^{\infty} k^2 p_k \\ &= \sum_{k=0}^{\infty} \frac{k^2 \epsilon \lambda^{\nu+k\tau}(1-\lambda^\tau)\beta^k \varphi^{kP}}{(1-\beta\lambda^\tau(\frac{1}{\beta}-\varphi^P))^{k+1}} \\ &= \frac{\epsilon\lambda^\nu(1-\lambda^\tau)}{1-\lambda^\tau(1-\beta\varphi^P)} \sum_{k=0}^{\infty} k^2 \left[\frac{\lambda^\tau\beta\varphi^P}{1-\lambda^\tau(1-\beta\varphi^P))}\right]^k . \end{aligned}$$

Using the sum of power series, that is $\sum_{n=0}^{\infty} n^2 x^n = \frac{x+x^2}{(1-x)^3}$ to simplify the terms not involving the summation sign, we have

$$\begin{aligned} M_2 &= \frac{\epsilon\lambda^\nu(1-\lambda^\tau)}{1-\lambda^\tau(1-\beta\varphi^P)} \left[\frac{\frac{\lambda^\tau\beta\varphi^P}{1-\lambda^\tau(1-\beta\varphi^P)} + (\frac{\lambda^\tau\beta\varphi^P}{1-\lambda^\tau(1-\beta\varphi^P)})^2}{(1-\frac{\lambda^\tau\beta\varphi^P}{1-\lambda^\tau(1-\beta\varphi^P)})^3}\right] \\ &= \frac{\epsilon\lambda^\nu(1-\lambda^\tau)}{1-\lambda^\tau(1-\beta\varphi^P)} \left[\frac{\frac{\lambda^\tau\beta\varphi^P(1-\lambda^\tau(1-\beta\varphi^P)))+(\lambda^\tau\beta\varphi^P)^2}{(1-\lambda^\tau(1-\beta\varphi^P))^2}}{\frac{(1-\lambda^\tau(1-\beta\varphi^P)-\lambda^\tau\beta\varphi^P)^3}{(1-\lambda^\tau(1-\beta\varphi^P))^3}}\right] \\ &= \epsilon\lambda^\nu(1-\lambda^\tau)\left[\frac{\lambda^\tau\beta\varphi^P(1-\lambda^\tau(1-\beta\varphi^P)+\lambda^\tau\beta\varphi^P)}{(1-\lambda^\tau)^3}\right] \\ &= \epsilon\lambda^\nu(1-\lambda^\tau)\left[\frac{\lambda^\tau\beta\varphi^P(1-\lambda^\tau(1-2\beta\lambda^\tau\varphi^P))}{(1-\lambda^\tau)^3}\right] \\ &= \frac{\epsilon\lambda^{\nu+\tau}\beta\varphi^P(1-\lambda^\tau(1-2\beta\varphi^P))}{(1-\lambda^\tau)^2}. \end{aligned}$$
