# Supplementary figures and images for: Improved estimates for extinction probabilities and times to extinction for populations of tsetse (Glossina spp)
Source: PLoS Negl Trop Dis. 2019 Apr 9;13(4):e0006973. doi: 10.1371/journal.pntd.0006973 (PMC6474634; doi:10.1371/journal.pntd.0006973)

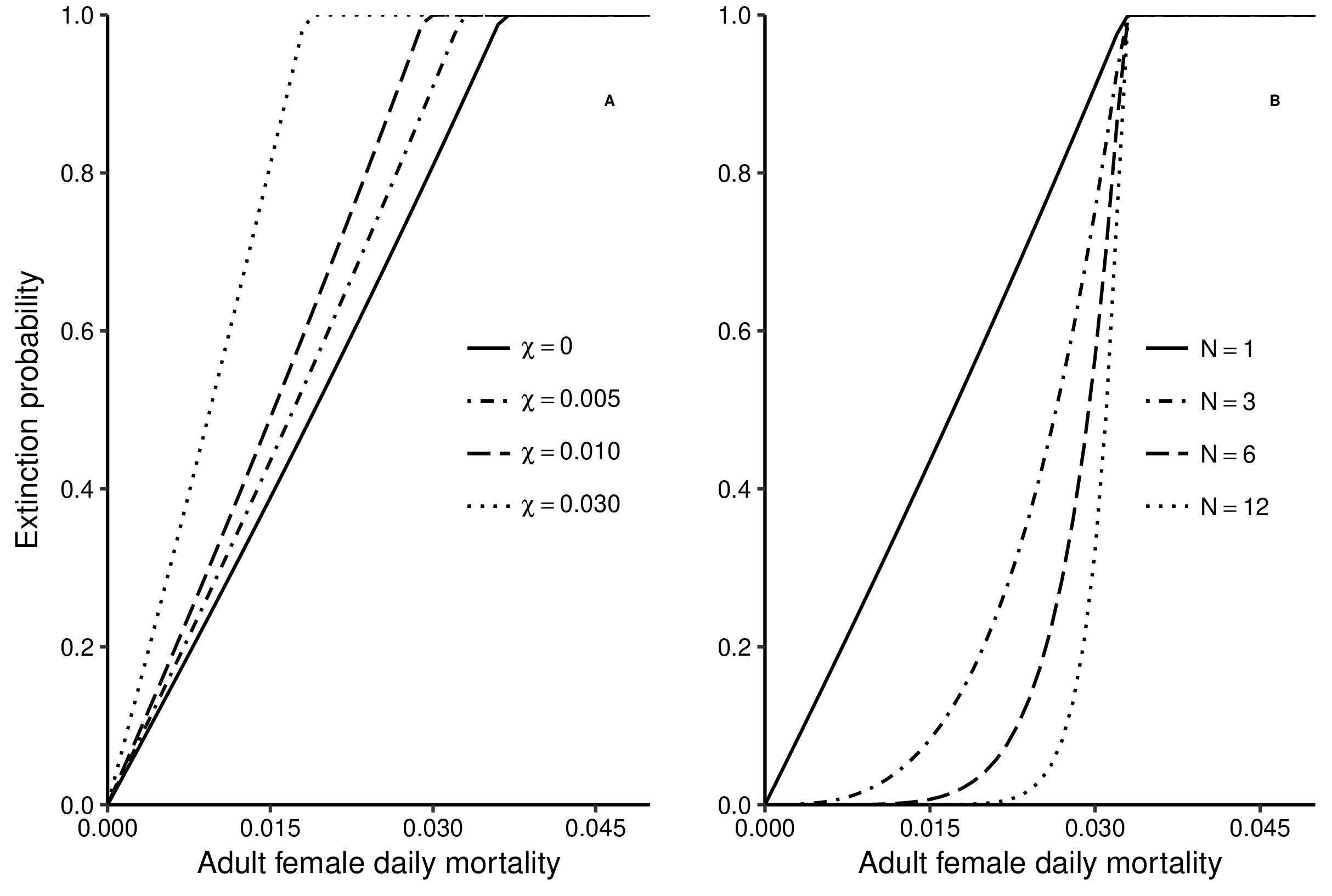

Supplement: S1 Fig — Input assumptions: Probability females inseminated by a fertile male, ϵ = 1.0; probability deposited pupa is female, β = 0.5; pupal duration, P = 27 days; time to first ovulation, ν = 7 days; inter-larval period τ = 9 days. A: Extinction probability as a function of female adult, and pupal, mortality rates. Extra input assumption: Pioneer population N = 1 inseminated female. Figures in the body of the plot show the assumed pupal mortality rate (χ per day). (cf [3], Fig 1A). B: Extinction probability as a function of adult female mortality rate and the number of inseminated females in the pioneer population. Extra input assumption: Pupal mortality rate assumed constant at a level of χ = 0.005 per day. Figures in the body of the plot show the assumed number (N) of inseminated females in the pioneer population. (cf [3], Fig 1B). (TIF) [file pntd.0006973.s002.tif]

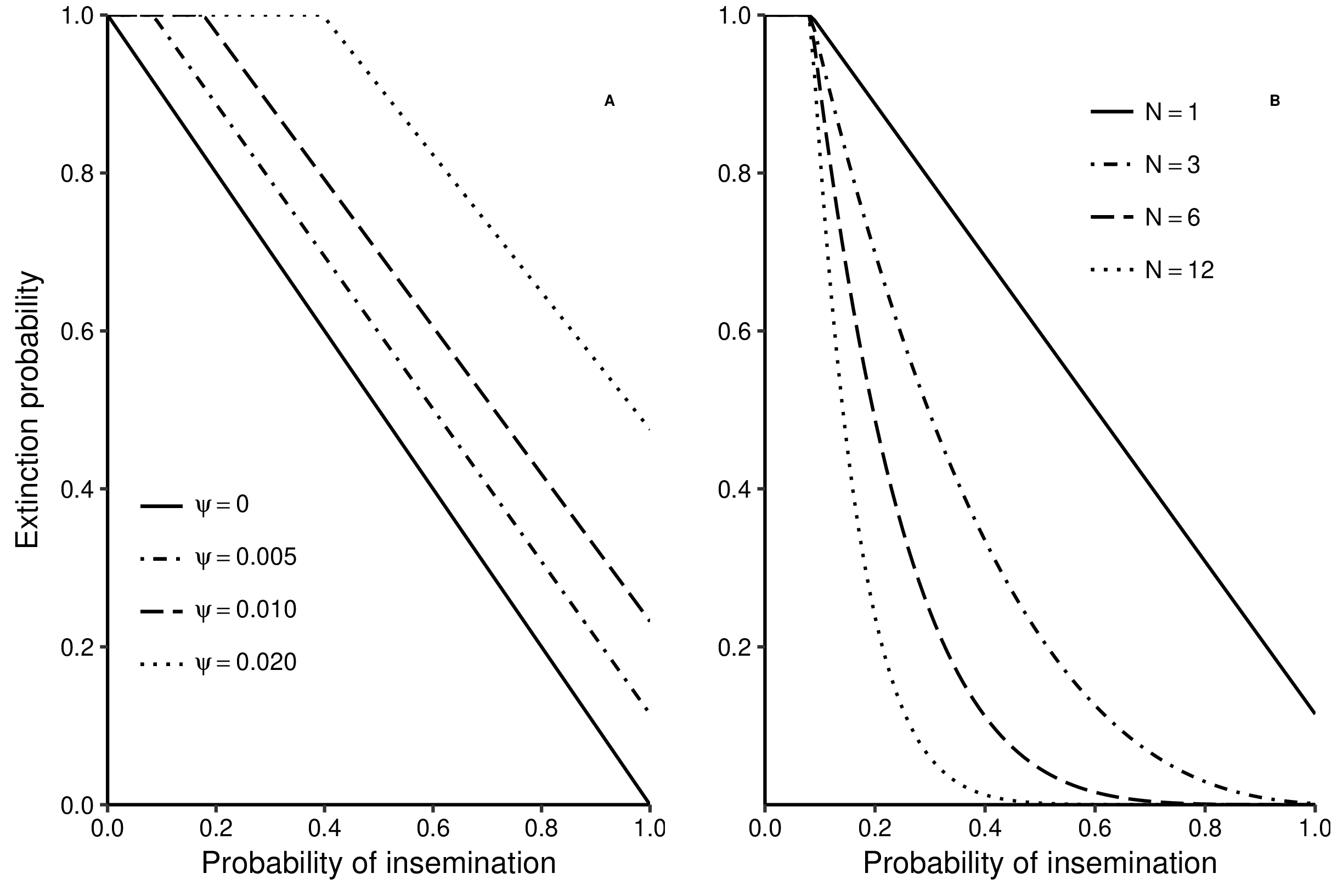

Supplement: S2 Fig — Input assumptions: Pupal mortality rate χ = 0.005 per day; probability deposited pupa is female, β = 0.5; pupal duration, P = 27 days; time to first ovulation, ν = 7 days; inter-larval period ν = 9 days. A: Extinction probability as a function of the probability that a female is inseminated by a fertile male, for different levels of adult female mortality rate. Extra input assumption: Pioneer population N = 1 inseminated female. Figures in the body of the plot show the assumed adult mortality rate (ψ per day).(cf [3], Fig 2A). B: Extinction probability as a function of the probability that a female is inseminated by a fertile male, and the number of inseminated females in the pioneer population. Extra input assumption: Female adult mortality rate assumed constant at a level of ψ = 0.005 per day. Figures in the body of the plot show the numbers (N) of inseminated females in the pioneer population. (cf [3], Fig 2B). (TIF) [file pntd.0006973.s003.tif]

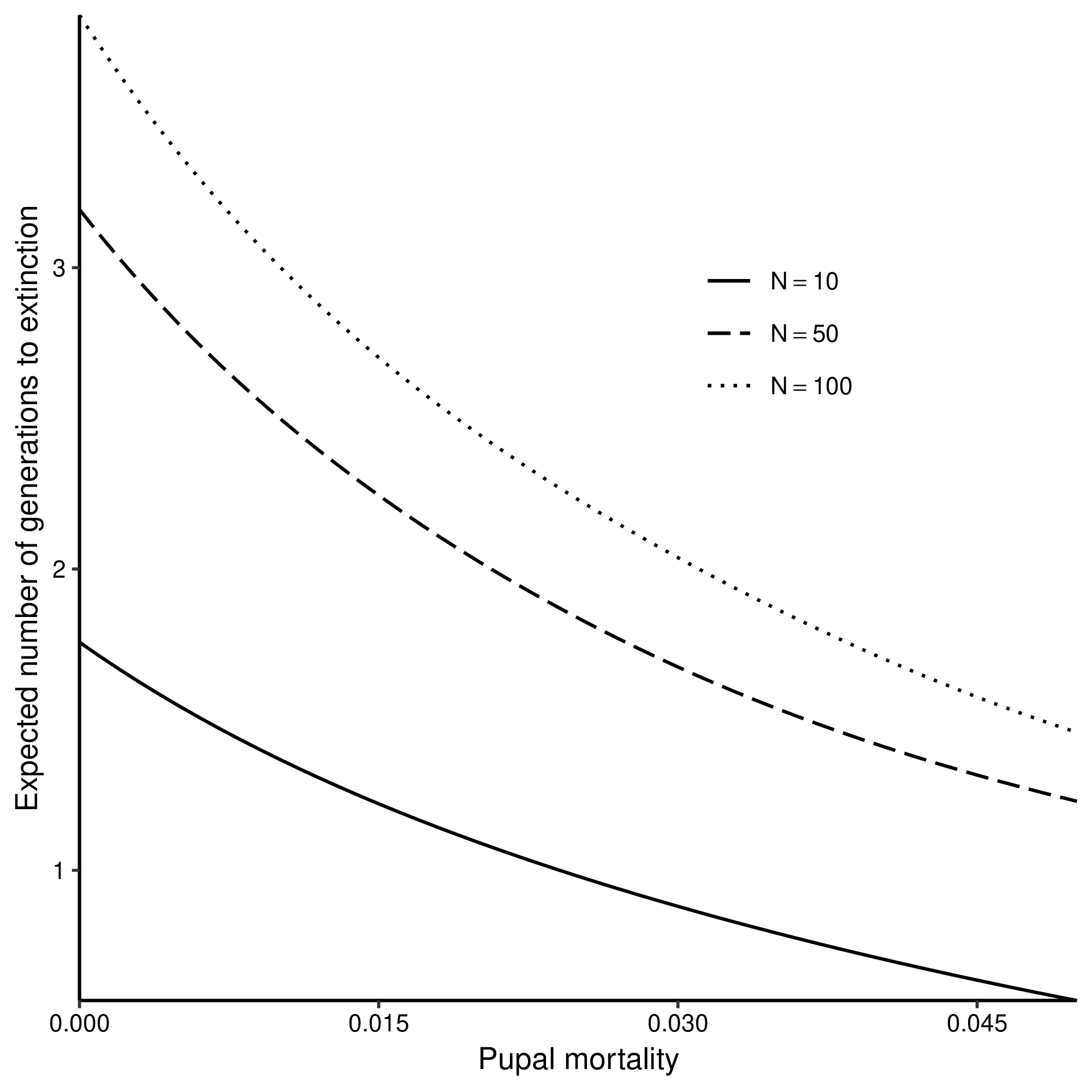

Supplement: S3 Fig — Input assumptions: Adult mortality rate ψ = 0.07 per day; probability deposited pupa is female, β = 0.5 probability females inseminated by a fertile male, ϵ = 1.0; pupal duration, P = 27 days; time to first ovulation, ν = 7 days; inter-larval period τ = 9 days. Figures in the body of the plot show the number (N) of inseminated females in the pioneer population. (cf [3], Fig 5A). (TIF) [file pntd.0006973.s004.tif]

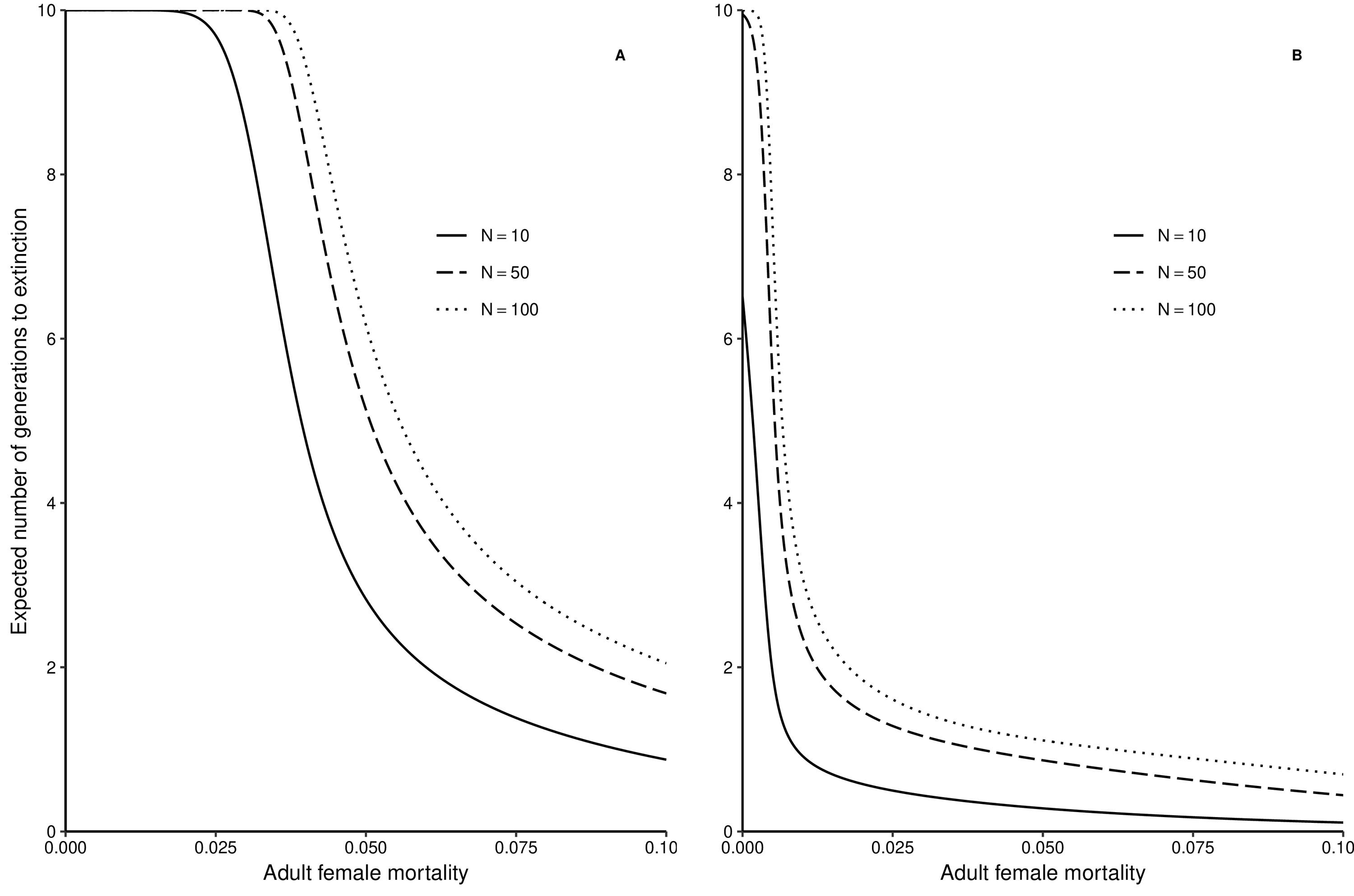

Supplement: S4 Fig — Input assumptions: Pupal mortality rate χ = 0.005 per day; probability deposited pupa is female, β = 0.5; pupal duration, P = 27 days; time to first ovulation, ν = 7; inter-larval period τ = 9 days. Figures in the body of the plot show the number (N) of inseminated females. (cf [3], Fig 5). A: Probability females inseminated by a fertile male, ϵ = 1. B: Probability females inseminated by a fertile male, ϵ = 0.1. (TIF) [file pntd.0006973.s005.tif]

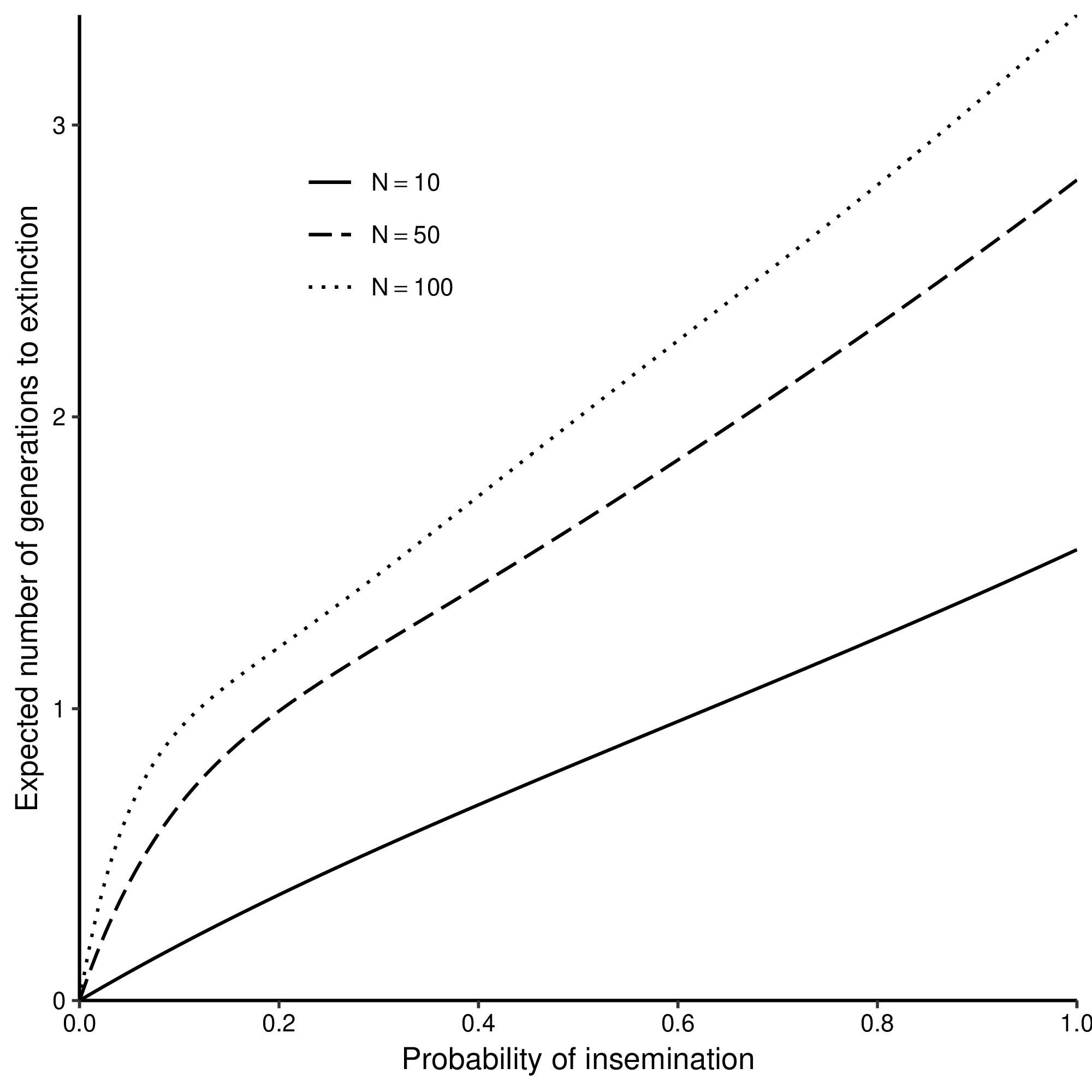

Supplement: S5 Fig — Input assumptions: Adult mortality rate ψ = 0.07 per day; pupal mortality rate χ = 0.005 per day; probability deposited pupa is female, β = 0.5; pupal duration, P = 27 days; time to first ovulation, ν = 7 days; inter-larval period τ = 9 days. Figures in the body of the plot show the number (N) of inseminated females (cf [3], Fig 5A). (TIF) [file pntd.0006973.s006.tif]
